# One-class modeling for verification of botanical identity: a review

**DOI:** 10.3389/fphar.2025.1504230

**Published:** 2025-03-27

**Authors:** James Harnly

**Affiliations:** Methods and Applications Food Composition Lab, Beltsville Human Nutrition Research Center, Agricultural Research Service, U.S. Department of Agriculture, Beltsville, MD, United States

**Keywords:** PCA, one-class modeling, review, authentication, multivariate analysis

## Abstract

One-class modeling is a supervised multivariate botanical identification method based on principal component analysis (PCA) that constructs a model based only on the characteristics of the reference samples and uses the Q statistic as a combined metric. Test samples are judged to be similar (authentic) if their combined metric falls within the model limits or different (adulterated or contaminated) if the metric falls outside the model limits. This review initially considers three major factors affecting identification: the number of variables (univariate versus multivariate), the number of classes (one-class versus multi-class), and the type of analysis (quantitative versus qualitative). Multivariate analysis is commonly used for identification, providing a broader coverage of the identity specifications of the samples. With a combined metric, multivariate methods are analogous to univariate methods. One-class modeling and multi-class modeling employ different approaches for identification with one-class modeling being more flexible. While most methods to date have had a quantitative basis, qualitative methods are possible. This review focuses on multivariate, one-class modeling based on PCA. Examples are presented for the application of one-class modeling to identification of American ginseng (*Panax quinquefolius*), *Echinacea purpurea*, Black Cohosh (*Actaea racemosa*), and Maca (*Lepidium meyenii*). These examples demonstrate the utility and flexibility of one-class modeling.

## Graphical Abstract

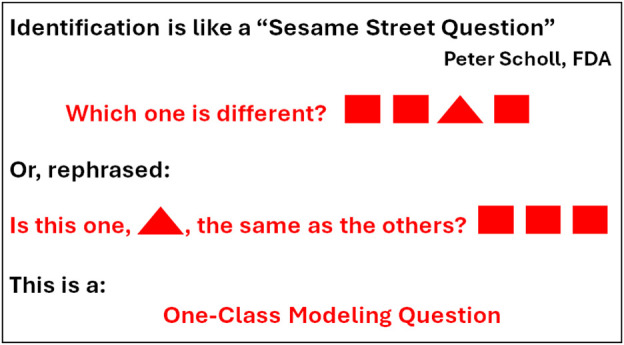

## Introduction

The goal of botanical identification is conceptually simple as shown by the graphical abstract; determine whether a test sample has features that are similar to those for a set of reference samples. This approach assumes that valid reference materials exist, and that identification can be made through a direct comparison of the test sample with a set of reference materials. The features used for a comparison can be sensory, morphological, microscopic, genetic, or chemical. The reference samples must be authenticated botanical reference materials or vouchered materials obtained from reliable sources. The authenticity of the reference materials is critical to the validity of the comparison. Multiple reference samples are needed to account for genetic, environmental, and processing variability. A single reference material from a metrological source can indicate similarity but fails to provide any information on biological variability.

Classically, herbalists, botanists, and taxonomists have authenticated plant materials based on monographs that describe a series of observations and tests developed using vouchered samples. In general, this approach requires access to the full plant material and flower. At one time, all commercial botanical materials were wild crafted by an expert and supplied directly to the user or to a local distributor. Today, botanical supplements are a multi-billion dollar industry and the link between the supplier and the manufacturer is much more tenuous. Manufacturers of botanical supplements are often faced with the problem of verifying that the barrel of brown powder delivered on the back dock by their supplier is really *ginkgo biloba*.

Commercial botanical supplements are classified as a food and are only regulated by FDA’s current good manufacturing practices (cGMPs) which require the manufacturer to describe the steps they took to test the purity of their ingredients and products (Food and Drug Administration and Health and Human Services, 2024). Since the ingredients have often lost their morphological integrity (e.g., they may be sold as powders or extracts), chemical methods are most frequently used for identification although genetic methods are becoming more popular with recent advances in technology. Chemical methods provide quantitative multivariate data in the form of chromatograms or spectra that can be used for targeted or non-targeted analysis (Nichani et al., 2023a; Nichani et al., 2023b). However, these methods can only be used for identification by comparison to appropriate reference materials.

The need for identification methods led AOAC International to develop “Guidelines for Validation of Botanical Identification Methods” in 2012 (Harnly, 2012). The Guidelines describe a probability of identification (POI) method, establish a well defined nomenclature (Table 1), and describe several basic principals. First, the guidelines recognize the use of both quantitative chemical methods and qualitative morphological methods. Second, the guidelines assume a multivariate analysis and specify that the chosen method must reduce multiple observations or measurements to a combined metric. Next, the metric is used to generate a binary response, “yes” the sample is authentic or ‘no” it is adulterated. Finally, the POI method is a two-class analysis method that requires comparison of an authentic and an adulterated sample (Table 2) (Brereton, 2009). Since the Guidelines are not method specific, 60 analyses of the authentic and adulterated samples are required to guarantee 95% confidence in discriminating between the two populations (Table 3). A companion paper to the Guidelines presented an example of a quantitative chemical analysis using mass spectrometric data (LaBudde and Harnly, 2012). Unfortunately, an example based on a qualitative morphological analysis was not given and has not been forth coming.

**TABLE 1:** Glossary (LaBudde and Harnly, 2012).

Botanical: Of, or relating to, plants or botany. May also include algae and fungi. May refer to the whole plant, a part of the plant (e.g., bark, woods, leaves, stems, roots, rhizomes, flowers, fruits, seeds, etc.), or an extract of the parts. Botanical identification method (BIM): A method that establishes identity specifications for a botanical material and determines YES, the test material is a true example of the target botanical material or NO, it is not the target botanical. Combined Metric: (analytical parameter in previous papers) A measured or computed analytical value used to determine whether the test material matches the target material. The combined metric may be based on morphological features, genetic sequences, chromatographic patterns, spectral patterns, or any other metric appropriate for the target material. Exclusivity Panel: A list of practically obtainable botanical materials that that are expected to give a negative result when tested by the BIM. Identity specification (IS): The morphological, genetic, chemical, or other characteristics that define a target botanical material. Specifications may include, but are not limited to, data from macroscopic, microscopic, genetic (e.g., DNA sequencing), chromatographic fingerprinting (e.g., capillary electrophoresis, gas chromatography, liquid chromatography, or thin-layer chromatography), and spectral fingerprinting (e.g., infrared, near-infrared, nuclear magnetic resonance, ultraviolet/visible absorbance, or mass spectrometry) methods. Inclusivity Panel: A list of practically obtainable botanical materials that are expected to give a positive result when tested by the BIM. Non-target botanical material: Any botanical material that does not meet the identity specification. Probability of identification (POI): The expected or observed fraction of test portions at a given concentration that give a positive result when tested by the BIM. Sample: A small portion or quantity, taken from a population or lot that is ideally a representative selection of the whole. Sensitivity: Ability of a BIM to correctly identify variants of the target material that meet the identity specification. Specificity: Ability of a BIM to correctly reject nontarget botanical materials. Standard inferior test material (SITM): A botanical material mixture that has the maximum concentration of target material that is considered unacceptable, as specified by the SMPRs. The BIM must reject this material. Standard method performance requirements (SMPRs): Performance requirements based on the fitness-for-purpose statement for each method. For BIMs, the SMPRs should include the physical form of the sample, the ISF, the ESF, the SSTM, the SITM, the number of samples for the inclusivity/ exclusivity panels, and the desired probability and confidence limits for the method. Standard superior test material (SSTM): A botanical material mixture that has the minimum acceptable concentration of the target material, as specified by the SMPR. The BIM must accept this material. Target botanical material: The botanical material of interest as described in the identity specification.

**TABLE 2:** Chemometric methods (Brereton, 2009).

Approach	Method^a^	Supervision	Classes
Exploratory	PCA	None	None
One Class Modeling (soft modeling)	PCA	ID 1 class	1
	SIMCA	ID all classes	2 or more
Two Class Classification (hard modeling)	LDA	ID all classes	2 or more
	QDA	ID all classes	2 or more
	PLS-DA	ID all classes	2 or more
	SVM	ID all classes	2 or more
	ANN	ID all classes	2 or more

^a^ ANN, Artificial neural networks; LDA, Linear discriminate analysis; PLS-DA, Partial least squares-discrimination analysis; PCA, Principal component analysis; QDA, Quadratic discriminate analysis; SIMCA, Soft independent modeling of class analogy; SVM, Support vector machines.

**TABLE 3:** Sample size required for proportion (Harnly, 2012).

Minimum	Number	Number	1-Sided	2-Sided	2-Sided	Effective
Probability	tests	failures	LCL	LCL	UCL	AOQL
50%	10	2	54.1%	49.0%	94.3%	71.7%
50%	20	6	51.6%	48.1%	85.5%	66.8%
50%	40	14	52.0%	49.5%	77.9%	63.7%
50%	80	32	50.8%	49.0%	70.0%	59.5%
55%	10	1	65.2%	59.6%	100%	79.8%
55%	20	5	56.8%	53.1%	88.8%	71.0%
55%	40	12	57.1%	54.6%	85.8%	68.2%
55%	80	28	55.9%	54.1%	74.5%	64.3%
60%	10	1	65.2%	59.6%	100%	79.8%
60%	20	4	62.2%	58.4%	91.9%	75.2%
60%	40	10	62.4%	59.8%	85.8%	72.8%
60%	80	24	61.0%	59.2%	78.9%	69.1%
65%	10	1	65.2%	59.6%	100%	79.8%
65%	20	3	67.8%	64.0%	94.8%	79.4%
65%	40	9	65.1%	62.5%	87.7%	75.1%
65%	80	21	65.0%	63.2%	82.1%	72.7%
70%	10	0	78.7%	72.2%	100%	86.1%
70%	20	2	73.8%	69.9%	97.2%	83.6%
70%	40	7	70.7%	68.0%	91.3%	79.7%
70%	80	17	70.4%	68.6%	86.3%	77.4%
75%	10	0	78.7%	72.2%	100%	86.1%
75%	20	1	80.4%	76.4%	100%	88.2%
75%	40	5	76.5%	73.9%	94.5%	84.2%
75%	80	13	75.9%	74.2%	90.3%	82.2%
80%	20	1	80.4%	76.4%	100%	88.2%
80%	40	3	82.7%	80.1%	98.6%	88.8%
80%	80	10	80.2%	78.5%	93.1%	85.8%
85%	20	0	88.1%	83.9%	100%	91.9%
85%	40	2	86.0%	83.5%	98.6%	91.1%
85%	80	6	86.1%	84.6%	96.5%	90.6%
90%	40	0	93.7%	91.2%	100%	95.6%
90%	60	2	90.4%	88.6%	99.1%	93.9%
90%	80	3	91.0%	89.5%	98.7%	94.1%
95%	60	0	95.7%	94.0%	100%	97.0%
95%	80	0	96.7%	95.4%	100%	97.7%
95%	90	1	95.2%	94.0%	100%	97.0%
98%	130	0	98.0%	97.1%	100%	98.6%
98%	240	1	98.2%	97.7%	100%	98.8%
99%	28/0	0	99.0%	98.6%	100%	99.3%
99%	400	1	99.1%	98.8%	100%	99.4%

Assume: 1. Binary outcome (occur/not occur).

2. Constant probability of event occurring

3. Independent trials

4. Fixed number of trials

Inference: 95% confidence interval lies entirely at or above the specified minimum.

Desired: Sample size N.

Notes: 1. Based on modified Wilson score 1-sided confidence limit.

2. AOQL = Average Outgoing Quality Level

It is recognized that the POI method is philosophically and statistically defensible but not practical. There have been very few applications of the POI method to identification problems (Harnly et al., 2013). Major obstacles are the need to identify each potential adulterant and to test each with 60 analyses. A daunting task. AOAC International has recently charged an expert panel with the development of new guidelines.

A new method has recently been proposed based on one-class modeling using principal component analysis (PCA) (Harnly et al., 2013; Harnly, 2023). This approach simplifies the identification process. It builds a single model based on the reference samples and treats every other sample as a potential adulterant. If an unknown sample falls within the model, the sample is judged to be authentic. If it falls outside the model it is deemed to be adulterated. The method is compatible with any multivariate data set and the Q statistic (Brereton, 2009) of PCA provides an inherent combined metric that can be used to determine the confidence limit of the model (Harnly et al., 2013). The validity of the model can be established by the cross validation. Thus, one-class modeling simplifies identification by eliminating the need to identify every potential adulterant, provides an inherent combined metric for each sample, provides confidence limits based on the number and quality of the reference samples, and provides a binary authentic/non-authentic output based on a statistical analysis.

This review will consider how chemical identification can be based on one or more features (univariate or multivariate), one or more classes of samples (one-class or two-class modeling), and quantitative or qualitative data. Regardless the number of features, classes, or nature of the data, the method generates a combined metric that produces a binary result, “yes” or “no,” with regards to authenticity (Harnly, 2012). This review will focus on the use of one-class modeling of multivariate quantitative data using PCA (Harnly, 2023). One-class modeling offers an inherently different approach from the POI model and two-class modeling and requires only the identification of the reference samples. In the last 10 years, this approach has been used to authenticate numerous raw botanicals and botanical supplements (Harnly et al., 2013; Harnly et al., 2016; Harnly and Upton, 2024; Harnly et al., 2017; Geng et al., 2020). Several examples of the application of one-class modeling to identification problems will be presented.

### Univariate versus multivariate analyses

Identification methods, as stated, generally employ multivariate analysis. Qualitative morphological methods are inherently multivariate since all the features of a plant are involved in identification. Quantitative chemical methods offer non-targeted multivariate analyses based on many chromatographic and spectral methods such as gas and liquid chromatography (GC and LC) and infrared spectrometry (IR), near IR spectrometry (NIR), mass spectrometry, and nuclear magnetic resonance spectrometry (NMR) (Nichani et al., 2023a; Nichani et al., 2023b). These methods may offer hundreds, even thousands (depending on resolution), of variables to characterize a sample. The many variables improve the chances of including important components of chemical identity or observing components that are not present (adulterants or contaminants) in the reference samples. Multivariate methods are less susceptible to fraud. Ideally, a botanical can be characterized with respect to hundreds of components with known structural and concentration variations that will allow the user to discriminate between similar species, samples with intentionally altered compositions, or material substitutes. However, incorporating a large number of variables in a binary decision (authentic or not authentic) is a challenge. Reduction of multiple variables into a combined metric offers the advantage of using classic univariate statistics to make this binary decision.

**FIGURE 1:**
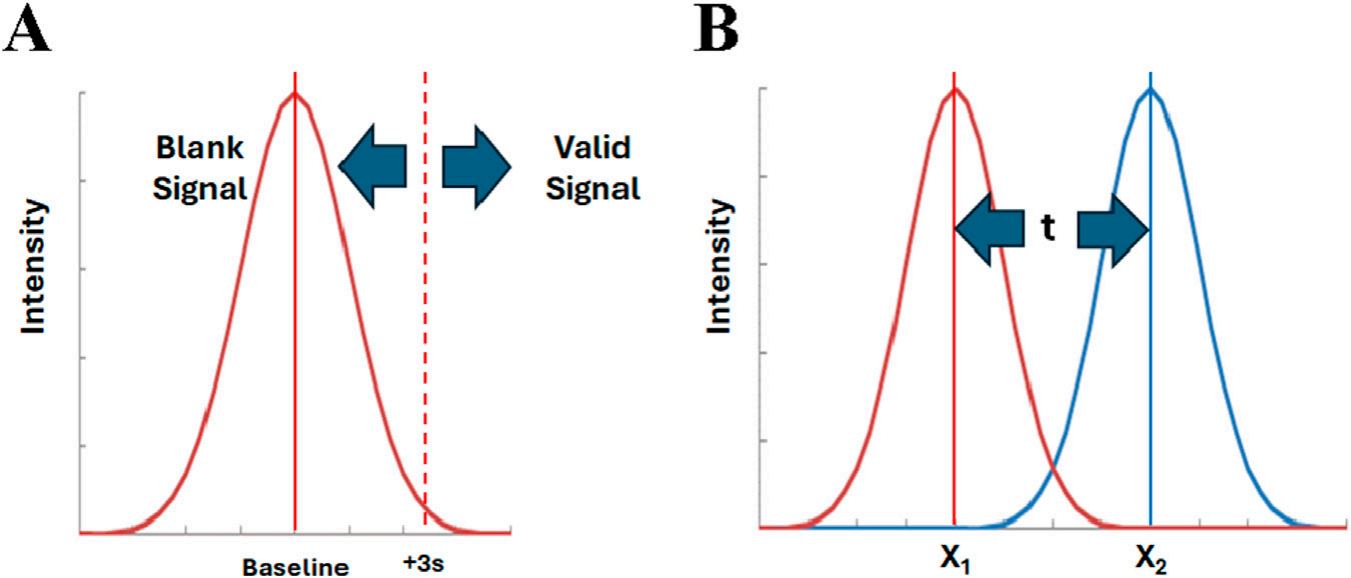
One class modeling and two class classification, **(A)** Detection limit and **(B)** Students’ t-test. Applied to univariate tests, the intensity is the measure for a single variable. For multivariate tests, the intensity is the combined metric computed from all variables.

There are two excellent examples that illustrate the use of univariate statistics for class analysis. First, the limit of detection (LOD) is a classic one-class model (Figure 1A) aimed at determining if a sample signal is a member of the blank signal population (Long and Winefordner, 1983). In the univariate mode, multiple measurements of the method blank establish the “baseline” (blank mean) and the standard deviation (distribution). Assuming a normal distribution, it is possible to statistically determine whether a signal is not simply due to random variation of the blank signal. For this purpose, a threshold is usually set at 3 times the baseline standard deviation (3s). This provides 99% confidence that any signal observed above this level is due to the presence of the analyte. Inversely, this threshold confirms that the test signal is not a member of the blank population. This is a form of one-class modeling.

Second, the students t test is a well established univariate approach to determining if the means of two populations are similar (Figure 1B) (Moore and McCabe, 1999). Multiple measurements of the value of interest are used to establish the mean and standard deviation of each population. Assuming both populations are normally distributed, the difference between the means of the populations is evaluated in terms of their standard deviations to establish the statistical significance. Similarly, the F-test can be used for establishing the difference for three or more populations (Moore and McCabe, 1999). These are forms of two-class or multi-class modeling. This approach is used as the basis of the POI method. However, in that case, the standard deviation is not known for either population, and the reference and adulterated samples must each be run 30 times to establish their distribution.

The diagrams in Figure 1 are the same for univariate and multivariate analyses, if the multivariate data is represented by a combined metric (Nichani et al., 2023a; Harnly, 2012; LaBudde and Harnly, 2012; Harnly et al., 2013). That is, all the variables from a multivariate analysis are used to compute a single, combined value or metric for each sample. This combined metric is then processed in exactly the same manner as a univariate value. The baseline value in Figure 1A corresponds to the combined metric mean of the reference samples. In this case, a test sample is judged to be authentic if it falls within the pre-determined level of uncertainty (e.g., within ±2s). In Figure 1B, the means correspond to the combined metrics for the reference population and the test (potentially adulterated) population. Classic statistics can be used to compute the confidence with which the two populations can be distinguished. When analyzed, the combined metric for the test sample will be judged to belong to either the authentic or test population.

For both the LOD and t-test, the number of measurements, n, for reference and test sample populations is critical to establishing the confidence with which similarity can be determined (Long and Winefordner, 1983; Moore and McCabe, 1999). The calculated mean and standard deviation of a population are only estimates of the true mean and standard deviation. As n increases, the level of confidence in the two values increases as does the decision regarding similarity.

### One-class modeling versus multi-class classification

There are numerous chemometric methods for processing multivariate data sets (Table 2). Unsupervised analysis requires no user input and reveals naturally occurring patterns. The most widely used unsupervised methods are PCA and hierarchical clustering analysis (HCA). Both serve to reveal sample patterns that may not be obvious. Supervised methods require identification of the classes of samples in the data set. Brereton has divided supervised multivariate analysis into the one-class classifiers and the two- (or multi-) class classifiers and described their fundamental differences (Brereton, 2009). Both approaches require a priori identification of the sample classes making them supervised methods.

One-class classifiers or one-class modeling constructs a model for each class based only on the characteristics of that class (Brereton, 2009). Comparison between sample classes requires comparison of the models for each class through soft independent modeling class analogy (SIMCA). SIMCA is considered a soft modeling technique as samples can be assigned to a single class, multiple classes, or no class. This is an excellent approach for recognizing outliers. Two-class classifiers or classification builds a single model for all the classes based on the features of all the classes (Brereton, 2009). Two-class classification is considered a hard modeling technique since samples are forced into one of the pre-designated classes. For example, partial least squares-discriminant analysis (PLS-DA) of *Gingko biloba* and *Echinacea purpurea* will require a sample of *Actaea racemosa* to be classified as either *Gingko* or *Echinacea*. Two-class classification methods have difficulty dealing with outliers and require re-calculation when additional classes are added.

One-class modeling has historically been used for process control (Brereton, 2009). Samples from key locations/times in a successful process are used to build models which can track the fidelity of succeeding processes. The one-class models tell the operator whether the process is within the limits established in previous runs.

**FIGURE 2:**
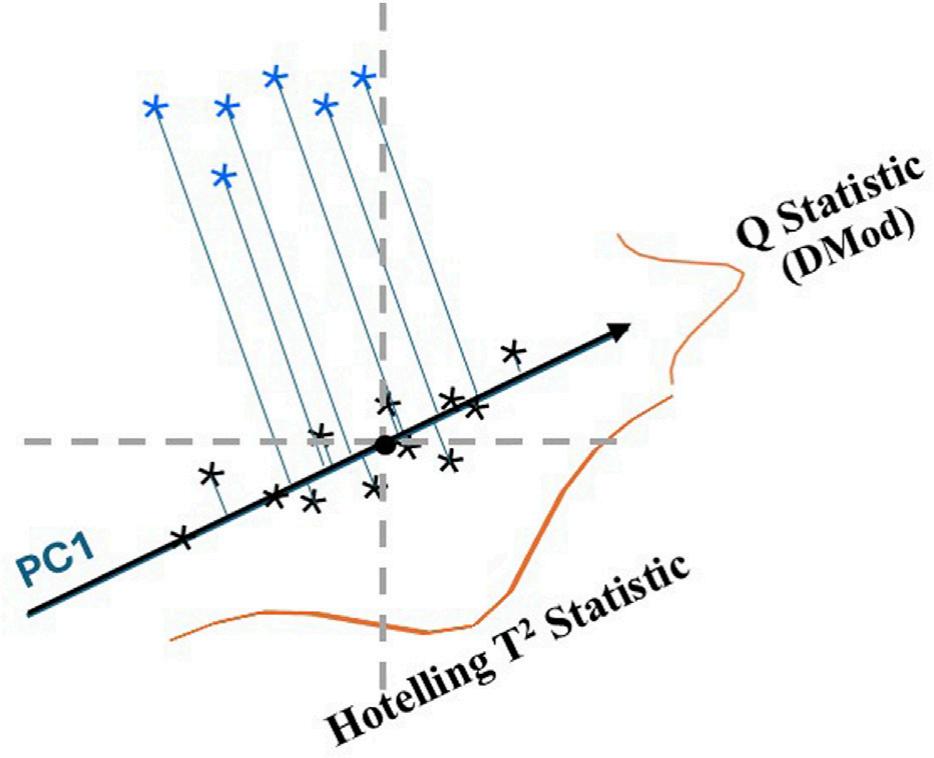
Illustration of the Hotelling T^2^ statistic and Q statistic for the first principal component (PC1) fit to a bivariate data set (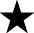) with test samples (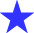). The Q statistic characterizes the variance outside the model, the distance from the sample perpendicular to the model, PC1. The Hotelling T^2^ statistic characterizes the variance within the model, the distance on PC1 from the sample intercept to the origin (mean centered data). The normal distribution of the Q statistic and the Hotelling T2 statistic are shown in red. A plot of the Q statistic versus the Hotelling T2 statistic is known as an influence plot.

There are numerous ways to compute a combined metric from multivariate analyses (Nichani et al., 2023a; Nichani et al., 2023b; Harnly, 2012; Brereton, 2009). The two statistical measures for evaluating a multivariate PCA model are shown in Figure 2 where a single principal component (PC1) is fit to a set of bivariate data (Brereton, 2009; LaBudde and Harnly, 2012). In this case PC1 is the model, it is a vector which intercepts the greatest variance of the data set (black symbols). The Q statistic is the variance outside the model, the distance of the sample from the model determined by a line from the sample perpendicular to the model. The Hotelling T^2^ statistic characterizes the sample variance within the model, the distance from the perpendicular intercept to the model center. This latter distance is analogous to the distance of a sample to the center of a normal distribution for univariate data. Figure 2 shows that the Hotelling T^2^ statistic would place the test samples (blue symbols) in the same class as the reference samples while the Q statistic shows that they belong in different classes. In general, the Q statistic is much more sensitive to compositional differences and detection of outliers.

One-class modeling using PCA is ideally suited for identification. A model can be built using reference samples, the Q statistic provides an excellent combined metric, confidence limits can be established, and a test sample can be determined to lie within (authentic) or outside (adulterated) the model. One-class modeling is flexible since only the reference data is identified. In the case of two classes of samples, both can be modeled and the models compared or either class can be modeled and the other treated as unknowns (see the example for black cohosh below).

## Quantitative versus qualitative

The facility of computing a combined metric from quantitative data using one-class modeling is readily seen in the preceding section. Both the Hotelling T^2^ statistic and the Q statistic are readily derived from chemometric analyses (Brereton, 2009) and provide a combined metric for all variables for each sample. Deriving such a metric for qualitative data is much more challenging (Harnly, 2012; Sudberg, 2024). The AOAC Guidelines state that they are intended for all candidate botanical identification methods and that identity specifications can be based on morphological, genetic, chemical, and/or other defining features of the botanical material. At that date it was envisioned that morphological or microscopic methods could be reduced to an algorithm or check list that would provide a suitable combined metric for judging authenticity. The companion paper (LaBudde and Harnly, 2012) illustrating the application of the POI method was based on quantitative analysis (mass spectral data) and, unfortunately, no example of a qualitative method was included.

There have not been any reports of qualitative identification in the literature. However, Sudberg (2024) at Alkemist Labs recently described a qualitative method for discriminating between spearmint (*Menta spicata* L.) and peppermint (*Mentha* x *piperita*) at the International Conference on Natural Products Research (Krakow, Poland, 2024) based on Bayes’ Theorem. This report nicely incorporated the POI principles which are discussed below.

### Probability of identification (POI)

The AOAC International POI method is based on two-class classification (Brereton, 2009). The glossary (Table 1) shows that the key feature of the method is to identify an authentic sample population that would always give a positive result (inclusivity sampling frame) and a population that would always give a negative result (exclusivity sampling frame). From their respective populations, a standard superior test material (SSTM, representing the minimum acceptable concentration of the target material) and standard inferior test material (SITM, representing the maximum unacceptable concentration of target material) are chosen to be run 30 times each to establish the precision of the method (Table 3).

In more general terms, an authentic (acceptable) and an adulterated (not acceptable) sample are chosen to characterize the resolution of the method, i.e., to determine if the method can discriminate between the two levels of adulteration. The adulterated sample is frequently an aliquot of the authentic sample spiked with a known level of adulterant or a different species from the same genus of the authentic sample (Nichani et al., 2023b; Harnly et al., 2013; Harnly, 2023; Harnly et al., 2016; Harnly and Upton, 2024; Harnly et al., 2017). Without specifying the method, the 30 repeats of each are used to establish the mean and distribution of the two populations (Harnly, 2012). The ability to perform 60 analyses with no false positives or negatives suggests a close to baseline separation of the two populations as shown in Figure 1B. Table 3 shows that 2 failures out of 60 translates to identification at the 90% confidence level 93.9% of the time, whereas no failures in 60 attempts corresponds to separation at the 95% confidence level 97.0% of the time.

The requirement of identifying an SITM for each potential adulterant and analyzing both the SSTM and SITM materials 30 times made the POI method unpopular. Today there is considerable interest in reducing the number of required analyses and expanding qualitative applications. However, it should be recognized that the number of samples analyzed, and the variance associated with the reference samples will always determine the confidence level that can be achieved by the method.

### One-class modeling

One-class modeling has been described in detail in a previous paper (Harnly et al., 2013) and in the previous section on “One-class modeling versus multi-class classification.” It makes use of the original requirement of the POI method to acquire an inclusivity panel (Table 2) of samples, requires establishing a PCA model, and uses the Q statistic as a combined metric to determine whether any test sample lies within or beyond the specified confidence limit of the model. There is no exclusivity panel, and any combination of unknown samples can be tested against the model. The false positive and negative rate are determined by the confidence level specified by the user. Validation of the model is established using either cross validation or an external validation sets of samples.

Like all statistical evaluations, the degree of confidence is dependent on n, the number of reference samples. In general, the more reference samples used, the more confidence the user has in the average Q value and the standard deviation. However, with respect to botanical materials, the biggest source of variability is the biological variability of the samples. Significant variability has been observed between sources, growing location, growing year, harvests, and from plant-to-plant. The more varied the plant meta-data, the greater the population distribution. As will be shown for the example of *A. racemosa* below, the variance covering three classes of identical BRMs from different sources is greater than the variance for each class.

**FIGURE 3:**
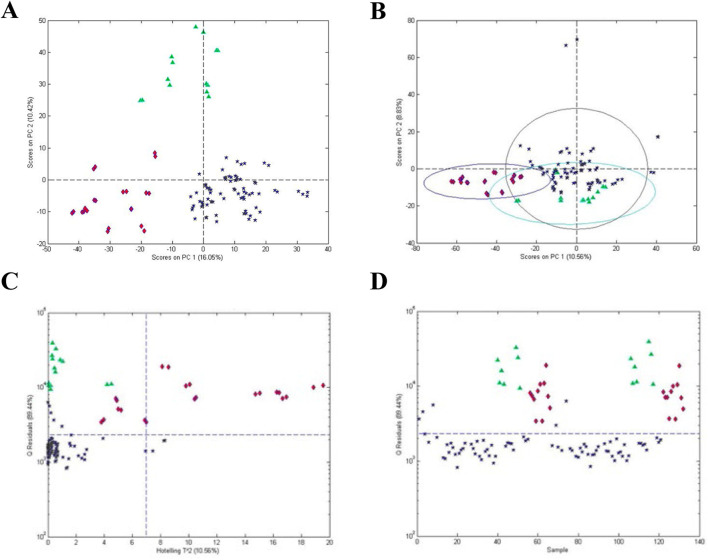
Analysis of processed Maca (*Lepidium meyenii*): **(A)** unsupervised PCA scores plot for all materials, **(B)** PCA scores plot based on one-class modeling of processed Maca from Peru, **(C)** influence plot (Q residuals versus Hotelling T2 residuals) based on one-class modeling of processed Maca from Peru, and **(D)** Q statistic versus sample based on one-class modeling of processed Maca from Peru. Symbols: (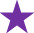) processed Maca from Peru, (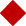) Maca root from Peru, and (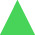) Maca root from China.

Figure 3 presents an example of the application of one-class modeling to a collection of Maca (*Lepidium meyenii*) samples consisting of roots collected in Peru and China and Processed Maca supplements from Peru (Geng et al., 2020). Processed root supplements are heated, extruded, powdered, and sold commercially. The question to be answered was could the processed Maca be distinguished from the root samples. Figure 3A shows an unsupervised PCA score plot for the Maca samples. The three clusters suggest the classes can be distinguished from each other but offer minimal statistics with respect to their differences. Figure 3B shows the results for supervised one class modeling of the processed Peruvian Maca samples. The variable loadings for the processed Maca samples were used to compute scores for the other samples (Peruvian and Chinese roots). Figure 3B shows ellipsoidal 95% confidence limits for each of the classes but it is again obvious that the score plot is a poor display for statistical differences of the classes.

Figure 3C presents a influence plot that displays the Q residuals (vertically) versus the Hotelling T^2^ residuals (horizontally) for a one class model of the processed Maca data, i.e., a influence plot for the data in Figure 3B. The data in Figure 3C were acquired using only one principal component, minimizing the possibility of over fitting. The statistics for the model can be seen much more clearly in the influence plot as can the difference between the Q and Hotelling T^2^ statistics. For the Hotelling T^2^ statistic, the sensitivity for the processed Maca is 97% and the specificity for the Peruvian and Chinese roots are 64% and 0%, respectively. For the Q statistic, the sensitivity is 93% and the specificity for both roots is 100%. Thus, the Q statistic is much more sensitive to differences in the sample composition. Plotting only the Q value versus individual samples (Figure 3D) presents a further simplified plot. This presentation can also be rotated to view the sample data as a frequency plot as will be shown below for one of the examples.

Choosing the processed Maca as the reference samples is arbitrary. Selection of the reference samples is dependent on the question being asked. The Peruvian or the Chinese root samples could also be chosen as the reference samples. The number of non-reference samples is also arbitrary. In Figure 3, two sets of non-reference samples (Peruvian and Chinese Maca roots) were chosen. Since the one-class model is based solely on the reference samples, the average and distribution of the non-reference samples is not a concern.

In essence, one-class modeling examines the difference associated with every variable in the data set (8). Simplistically, a model based on a mass spectrum of 205 ions requires that all 205 variables for the test material lie within the statistical limits determined for each variable of the reference samples. Practically, some deviation of variable(s) can be tolerated as determined by the limits set by the user. If a test material is to be judged different or adulterated, one (or more) of the variables in the data set must be significantly different. As shown in a previous study (Harnly, 2023), variables that are autoscaled will have a variance of 1.0. The 205 MS variables will have a total variance of approximately 205. The signal necessary for a variable to have a significantly different variance and have a significant statistical impact on the combined metric can be predicted from the average intensity of the variable (Harnly, 2023).

An added factor of considerable importance is that the plots in Figure 3 can be constructed by anyone using any commercial chemometric platform. Starting with the same data set and using the same pre-processing steps, any commercial platform will produce the same plots. Identification using one-class modeling can be done in any lab without the need for a chemometrics expert.

### Examples of one-class modeling

#### American Ginseng (*Panax quinquefolius*)

American Ginseng (PQ) adulterated with varying levels of Asian Ginseng (*Panax ginseng*, or PG) was used to illustrate an application of the POI (Harnly et al., 2013). For the study, 44 PQ samples were obtained from the Wisconsin Ginseng Board (harvested over 3 years from 20 different farms in Wisconsin) and 8 PG samples grown in China and acquired from American Herbal Pharmacopoeia and commercial sources. Samples were analyzed by flow injection mass spectrometry (FIMS) which yielded spectra with more than 1,000 ions.

**FIGURE 4:**
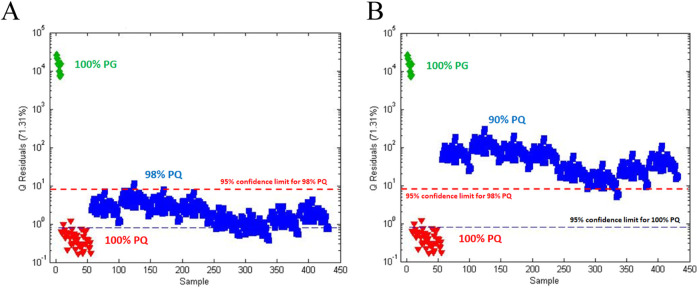
Q statistics plot of American ginseng (PQ) adulterated with Asian ginseng (PG) based on one-class modeling of 100% PQ: **(A)** Q statistic plot for (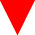) 100% PQ, (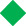) 100% PG, and (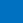) 98% PQ; **(B)** Q statistic plot for (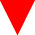) 100% PQ, (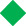) 100% PG, and (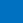) 90% PQ. The black dashed line is the 95% confidence limit for a one-class model of 100% PQ. The red dashed line is the 95% confidence limit for the one-class model of 98% PQ. Sensitivity for 100% and 98% PQ is 95% and 98%, respectively. Specificity for 100% and 90% PG is 100% and 99%, respectively.

The SSTM and SITM (Table 1) for the POI analysis were chosen as 98% and 90% PQ, respectively. Spectra for the test materials were synthesized mathematically using appropriate ratios of the 100% PQ and 100% PG spectra. In all, 344 (43 PQ × 8 PG) spectra were generated for each test material. The unsupervised PCA score plot (not shown) showed visual separation but the computed confidence limits did not allow detailed statistical analysis. Ironically, one-class modeling was used to provide detailed statistical analysis of the data although it was not the focus of the paper. The Q statistic served as the combined metric (Figure 4).

Figure 4A shows the Q statistic plot for 100% PQ, 100% PG, and 98% PQ with confidence limits based on one-class modeling for both 100% PQ and 98% PQ. The one-class model for 100% PQ has a sensitivity of 95% (42/44) and a specificity of 100%. The one-class model for 98% PQ, the SSTM, has a sensitivity of 98% (342/344) and a specificity of 100%. Figure 4B shows a similar plot with 90% PQ, the SITM. Based on the one-class model for 98% PQ, the 90% PG has a specificity of 99% (341/344).

**FIGURE 5:**
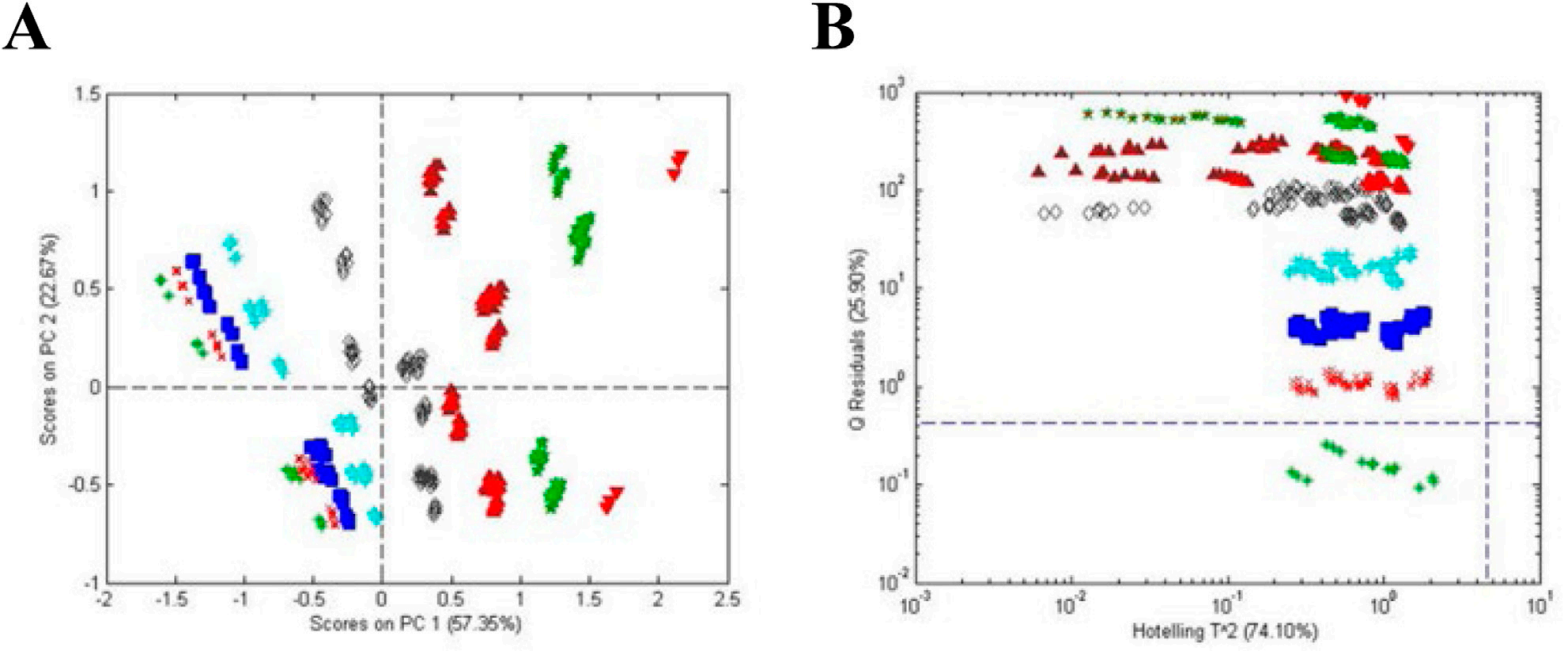
Adulteration of American ginseng (*Panax quinquefolius*) with Asian ginseng (*P. ginseng*): **(A)** Unsupervised PCA and **(B)** influence plot (Q residuals versus Hotelling T^2^ residuals) based on one-class modeling of 100% *P. quinquefolius*. Symbols: (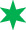) 100%, (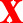) 95%, (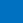) 90%, (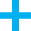) 80%, (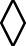) 60%, (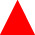) 40%. (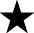) 20%, and (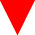) 0% PQ.

A follow up study verified the previous numerical dilution model with physical dilution of 100% PQ with 100% PG (Harnly et al., 2013). The more labor intensive nature of the physical dilutions restricted the number of samples. Five samples of 100% PQ (arbitrarily selected from the samples in the previous study) were diluted with two samples of PG at ratios of 95:5, 9:1, 8:2, 6:4, 4:6, and 2:8. Figure 5A shows that unsupervised PCA provided a near linear progression from 100% PQ to 100% PG on the X-axis indicating that PQ concentration was the primary source of variance. Figure 5B shows the influence plot (Q statistic versus Hotelling T^2^) based on a one-class model of 100% PQ. While the Hotelling T2 residuals failed to discriminate between the different PQ purities, the Q statistic provided excellent separation of the five PQ concentrations. A plot of the square root of the Q statistic showed a linear relationship with the PQ concentration (plot not shown).

**FIGURE 6:**
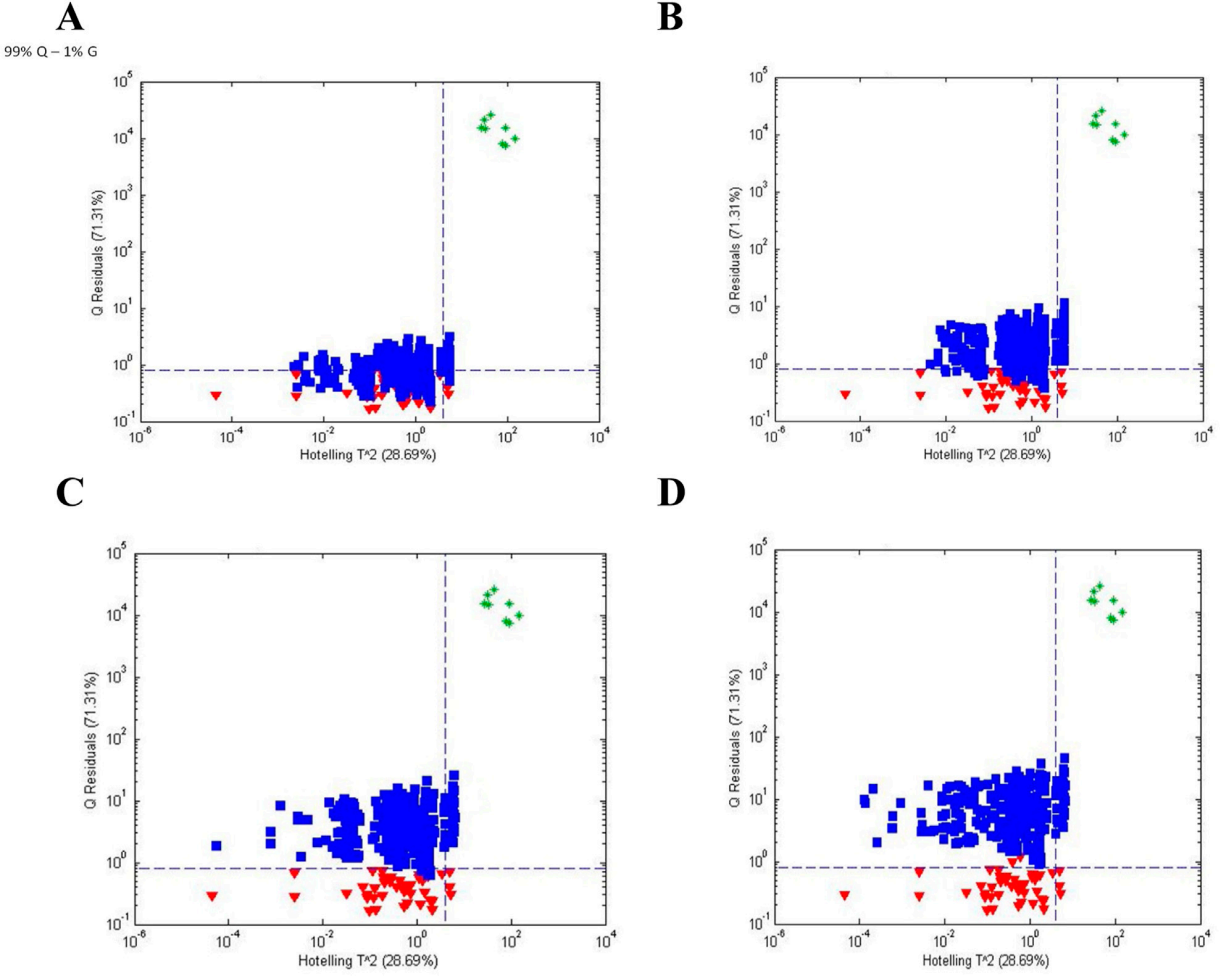
Influence plots (Q residuals versus Hotelling T ^ 2 residuals) for **(A)** PQ:PG = 99:1, **(B)** PQ:PG = 98:2, **(C)** PQ:PG = 97:3, and **(D)** PQ:PG = 96:4 based on one-class modeling of 100% PQ. Symbols: (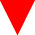) 100% PQ and (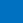) 99%, 98%, 97%, and 96% PQ.

An additional follow up study examined the ability of FIMS to discriminate between PQ:PG ratios of 99:1, 98:2, 97:3, and 96:4 (unpublished data) to determine the level of adulteration of PQ that could be detected. Figure 6 shows the influence plots (Q statistic versus Hotelling T^2^) for the 4 purity levels of PQ. Once again, Q statistic was more useful than Hotelling T^2^ for discriminating between populations. The sensitivity for 100% PQ was 95% and the specificity was 57%, 90%, 99%, and 100% for PG contamination of 1%, 2%, 3%, and 4%, respectively.

This study demonstrates the utility of one-class modeling for detecting adulteration. The Q values for physical and mathematical dilution showed excellent agreement. The digital model, based on the pure spectra (100%) of the authentic material and the adulterant, provides a much simpler means of evaluating the ability of the method to detect adulteration.

The POI studies used the term SIMCA (soft independent modeling of class analogy) instead of the one-class modeling. SIMCA, as explained earlier, consists of a series of one-class models. In all the studies reported in this paper, only a single class was used for modeling, a single PCA model. For the ginseng studies and all those that follow, the 95% confidence limit was chosen as the statistical limit. Other statistical limits can be selected by researchers. The Q and Hotelling T^2^ statistics were based on only one principal component, thus mitigating the possibility of over fitting. In addition, pre-processing for all the PCA calculations consisted of unit vector normalization of the samples (i.e., the sum of the squares of the sample intensities are set to 1.0) and autoscaling (normalization of each variable by its standard deviation) with mean centering of each variable.

#### Black cohosh (*Actaea racemosa*)

One-class modeling was used to distinguish *A. racemosa* L. (Ranunculaceae) from other *Actaea* species and commercially available roots and supplements (Harnly et al., 2016). FIMS and proton nuclear magnetic resonance spectrometry (NMR), two metabolic fingerprinting methods, and DNA sequencing were used to identify and authenticate the *A. racemosa* species. For this study, authentic *A. racemosa* botanical reference materials were acquired from four sources: American Herbal Pharmacopoeia (AHP), Strategic Sourcing (SS), North Carolina Arboretum (NCA), and the National Institutes of Standards and Technology (NIST). The NCA samples were triplicate samples collected from 22 sites across the eastern US.

**FIGURE 7:**
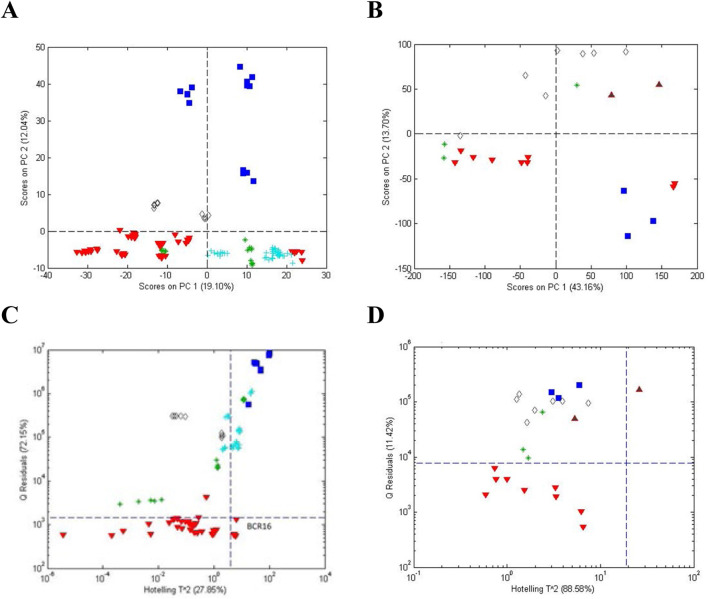
Unsupervised PCA scores plots **(A, B)** and influence plots (Q residuals versus Hotelling T^2^ residuals) **(C, D)** for one-class modeling of *A. racemosa* analyzed by FIMS **(A, C)** and NMR **(B, D)**. Symbols: (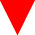) *A. racemosa*, (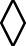) *A. rubra*, (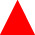) *A. cimicifuga*, (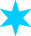) *A. pachypoda, and* (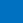) *A. podocarpa*.

Initial analyses of the *Actaea* species furnished by AHP using FIMS and NMR gave similar result as shown in Figure 7. PCA score plots (Figures 7A, B) for both methods showed a pattern differentiating *A. racemosa* from the other *Actaea* species. This differentiation was statistically verified by one-class modeling based on *A. racemosa* (Figures 7C, D). DNA sequencing using two independent gene regions (ITS and psbA-trnH) confirmed the metabolic fingerprinting results. Although not the point of this review, DNA sequencing provided identification for root materials but was inconsistent for the supplements. It was assumed that processing of the supplements destroyed the DNA in some cases.

**FIGURE 8:**
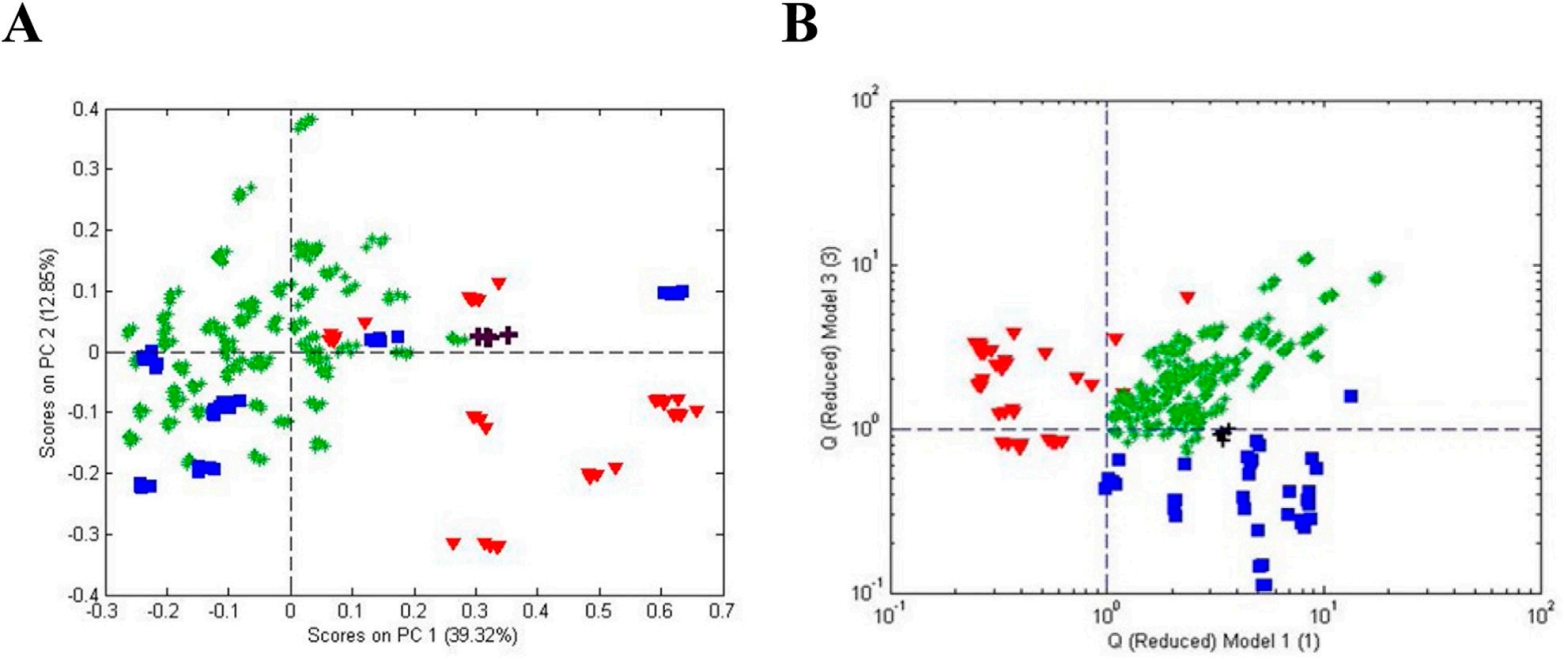
Comparison of *A. racemosa* reference samples from 4 sources (AHP, SS, NCA, and NIST). **(A)** Unsupervised PCA scores plot and **(B)** influence plot based on one-class modeling of *A. racemosa* from AHP (vertically) and SS (horizontally). Symbols: (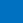) AHP, (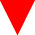) SS, (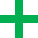) NIST, and (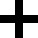) NCA. AHP and SS provided clusters below and to the left of the 95% confidence limits, respectively. NIST samples lay barely below the confidence limit with the AHP samples and NCA samples were outliers (upper right quadrant) for both one-class models.

The simplicity of categorization in Figure 7 was complicated when *A. racemosa* reference samples from the four sources were compared. A PCA score plot (Figure 8A) and a influence plot (combined one-class model plots for AHP and SS reference materials) (Figure 8B) demonstrated that the reference materials were statistically different. The plots show three separate groups with the NIST samples in the same quadrant as those from SS. Sensitivity for the AHP and SS samples was 94% and 91%, respectively, and 91% for the un-modeled NCA samples in the upper right quadrant, exceeding the 95% confidence limits for AHP and SS, i.e., outliers compared to the other reference materials. Interestingly, the NCA samples collected from 22 sites across the US showed a similar lack of uniformity. These data suggested that sample handling and storage, local environmental conditions, and/or genetic drift influenced the plant metabolic profiles. It was also suggested that endophytic bacteria might have a significant influence on the metabolic profile.

**FIGURE 9:**
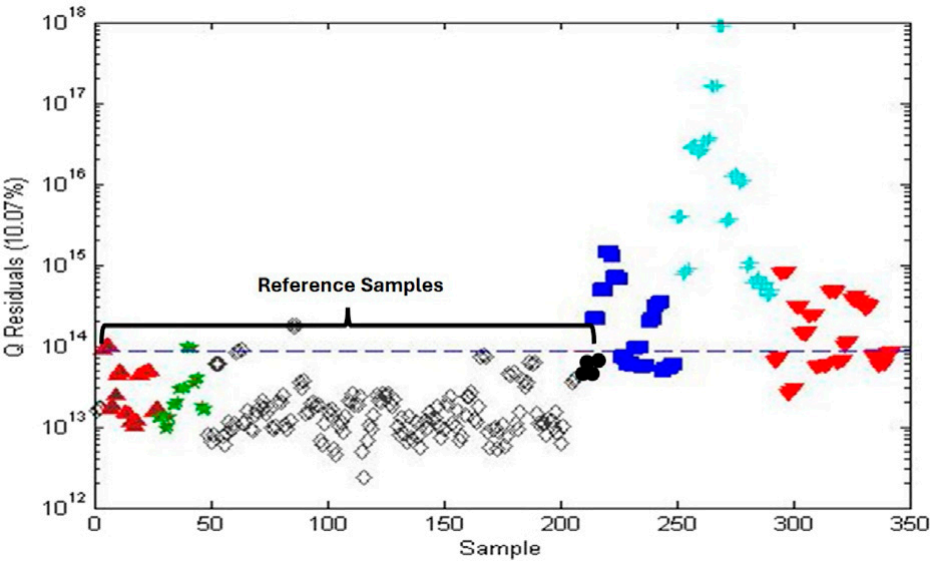
Q residuals plot based on one-class modeling of *A*. *racemosa* reference samples obtained from (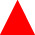) American Herbal Pharmacopoeia, (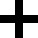) Strategic Sourcing, (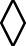) North Carolina Arboretum, and (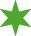) the National Institutes of Health. Non-modeled samples are: (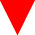) other *Actaea* species from AHP, (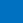) commercial *Actaea* roots, and (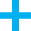) commercial *Actaea* supplements. The horizontal dashed line provides the 95% confidence limit, p = 0.05).

A follow-up study took a closer look at the differences between the reference samples and the effect of pre-processing on PCA and one-class modeling patterns (Harnly and Upton, 2024). In Figure 9, samples from all four sources were combined to serve as reference samples and used for one-class modeling. The variance of the combined reference materials exceeded that of each modeled individually, i.e., the model for AHP in Figure 7C. Sensitivity was 97% for the combined *A. racemosa* samples and the specificity was 56% for other *Actaea* species, 55% for commercial *Actaea* roots, and 100% for commercial *Actaea* supplements. These data demonstrated the difficulty of differentiating between the *Actaea* species and that some of the commercial root samples are correctly identified as *A. racemosa*. None of the commercial *Actaea* supplements exhibited metabolic profiles similar to the reference samples, most likely due to the influence of sample preparation.

**FIGURE 10:**
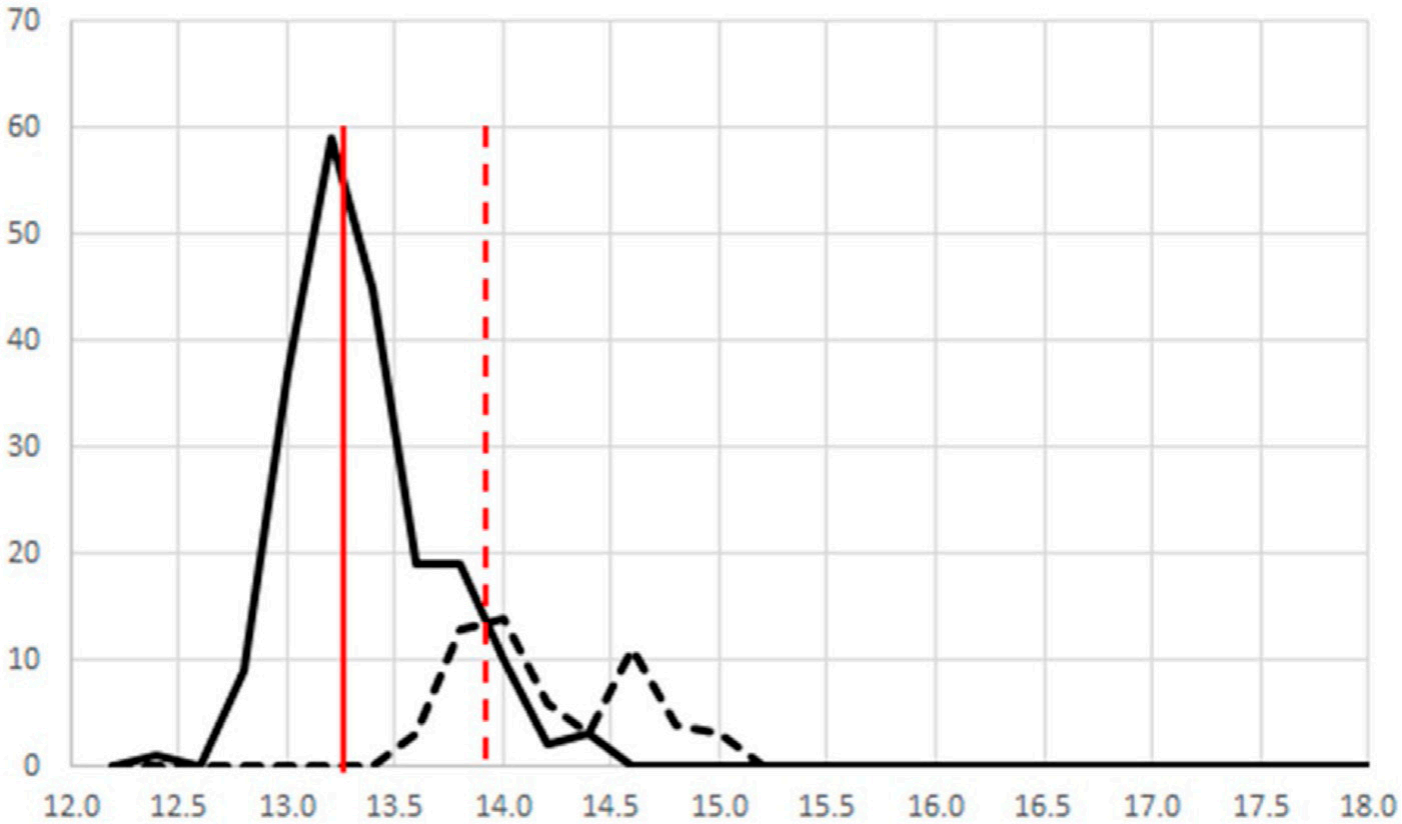
A side view of Figure 6 showing the frequency plot for (—) all 4 *A. racemosa* reference samples and (----) other *Actaea* species. The solid vertical line presents the mean for the *A. racemosa* reference samples and the dashed vertical line presents the +2s limit.

Figure 10 shows an alternate perspective for one-class modeling data. If the modeled data in Figure 9 are viewed from the side, they can be displayed as frequency plots. This is a more intuitive view, providing the mean and distribution of the populations. Figure 10 shows only the frequency plots for *A*. *racemosa* and the other *Actae*a species. Alternate pre-processing methods had little impact on the separation of the two populations. Use of only variables with a low variance provided improved overlap of the *A. racemosa* from the different sources (data not shown) but reduced the ability to discriminate between species (data not shown). Use of variables that enhanced discrimination between species (based on an f-test) also failed to enhance differentiation as the differences between the reference samples from the 4 different sources were also increased, i.e., the frequency plot for *A. racemosa* broadened (data not shown).

This study demonstrated that not all reference materials are created equal, use of multiple sources for reference materials increases the variance of the model, discriminating between species is difficult because the same metabolites are present but in a slightly different ratios, and finally, there is no simple pre-processing for improving discrimination between species.

#### Echinacea (*Echinacea purpurea*)

Preparation of commercial supplements from botanical ingredients results in changes in the chemical composition of the supplement. This makes it difficult to authenticate supplements based on the raw ingredients. One approach to comparing the composition of different samples is cross-correlation of the two spectra (Harnly et al., 2017). Ions found in both the reference samples and supplements spectra will provide an enhanced numerical product while those missing from either will yield a product close to zero. Comparison of the cross-correlated spectra can be achieved using PCA and eliminates the need for a normalization factor. As usual, however, one-class modeling provides a clearer analysis with statistical evaluation.

*E. purpurea* (L.) Moench aerial samples were chosen as the test material and cross-correlation and one-class modeling were used to distinguish *E. purpurea* (L.) aerial samples from *E. purpurea* and *Echinacea angustifolia* roots and supplements. Authentic *E. purpurea* aerial and root samples and *E. angustifolia* root samples were obtained from Missouri Botanical Gardens, American Herbal Pharmacopoeia, and commercial sources. Twenty-three liquid and solid (tablets and capsules) supplements were purchased locally; 11 were single-ingredient supplements containing only *E. purpurea* and *E. angustifolia* aerial or root material and 13 were mixed or unknown *Echinacea* supplements labeled to contain mixtures of *E. purpurea* with *E. angustifolia* and/or *Echinacea pallida* (Nutt.) and consisting of either aerial or root material. Samples were analyzed by FIMS.

**FIGURE 11:**
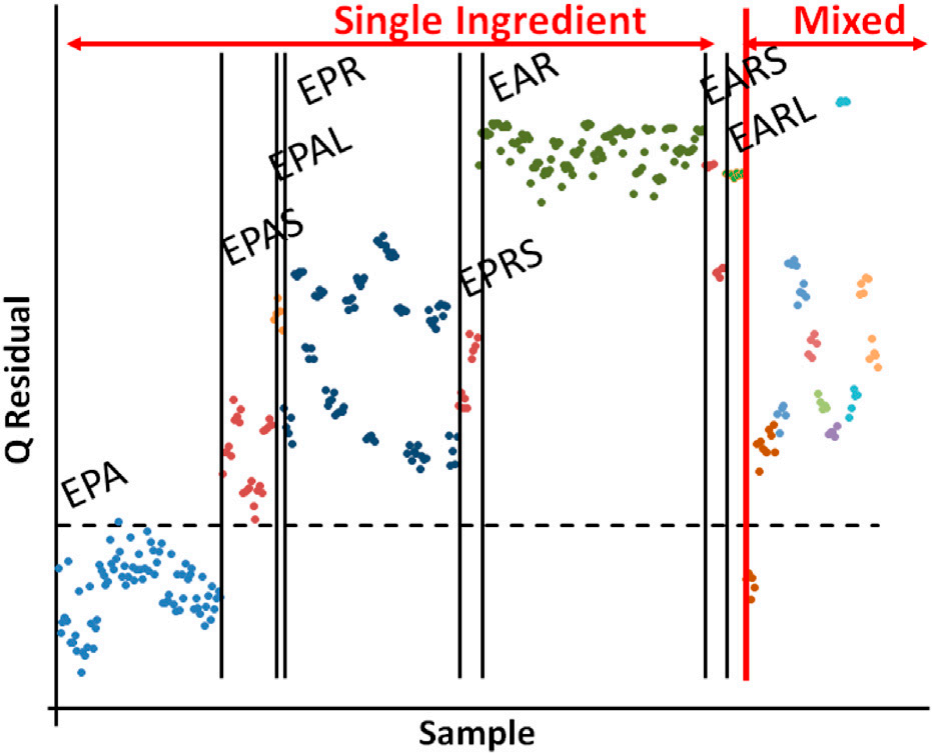
A Q statistics plot based on one-class modeling of *Echinacea purpurea* aerial samples (lower left). Labels for single ingredient samples: EPA – *E. purpurea* aerial samples, EPAS – EPA solid supplements, EPAL - EPA liquid supplements, EPR - E. purpurea root samples, EPRS - *E. purpurea* root solid supplements, EAR – *E. angustifolia* root samples, EARS - *E. angustifolia* root solid supplements, and EARL - E. angustifolia root liquid supplements. Mixed supplements (far right) are not individually labeled.

One-class modeling based on *E. purpurea* aerial samples showed that all other species and plant parts were significantly different from *E. purpurea* aerial (Figure 11). Thus, the compositional profiles of *E. purpurea* aerial could be differentiated from that of the *E. purpurea* roots and *E. angustifolia* roots and from any of the single ingredient or mixed supplements. The sensitivity for the model in Figure 11 was 99% and the specificity was 98%. These data reaffirm previous observations that most raw materials are not suitable for authenticating supplements. The preparation process for commercial samples (e.g., extraction, back extraction, drying, powdering, and possible addition of other materials) can result in significant differences in the chemical composition and their levels. It should be noted that his does not necessarily reflect on the supplements efficacy.

**FIGURE 12:**
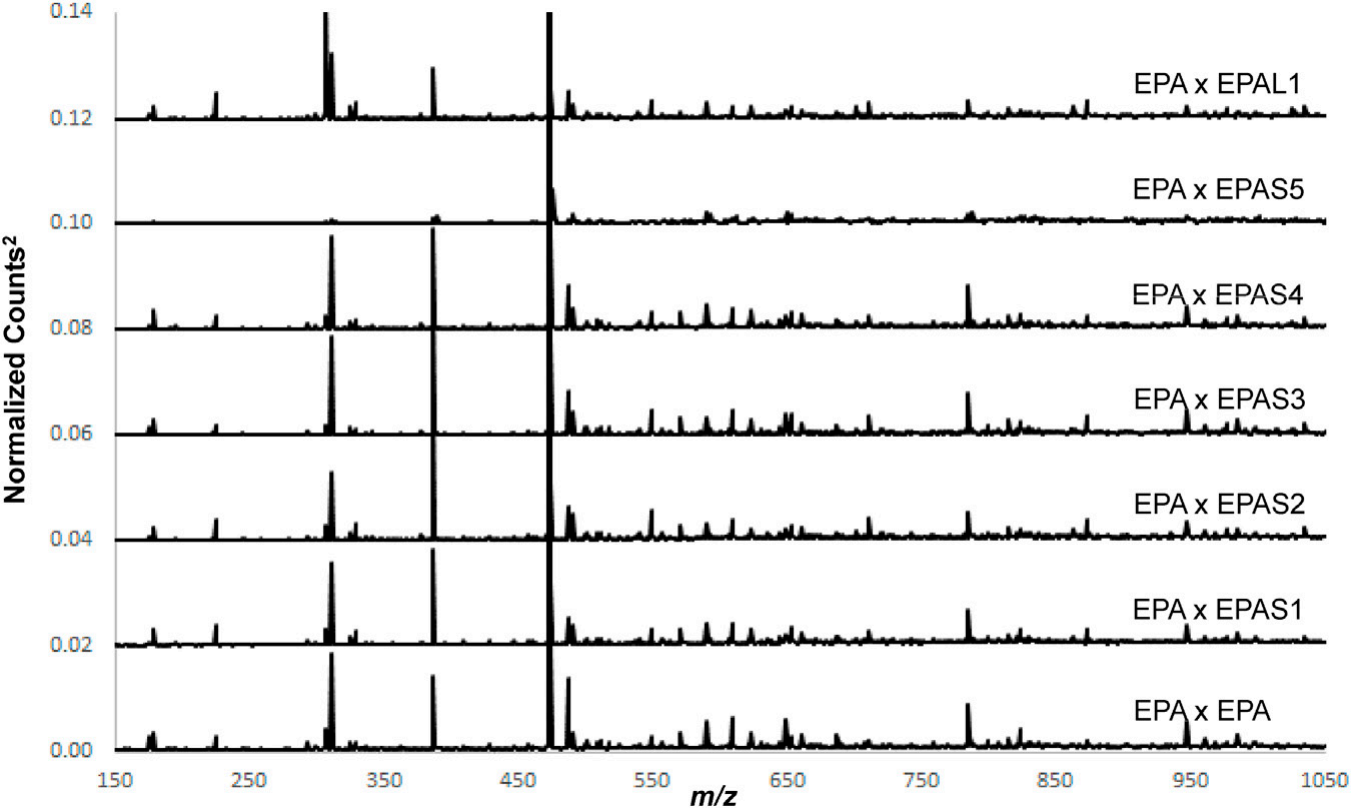
Spectra of *E. purpurea aerial* (EPA) cross-correlated with itself, *E. purpurea* aerial solid supplements 1–5 (EPAS1-EPAS5), and *E. purpurea* aerial liquid supplement sample (EPAL1).

Figure 12 shows the average EPA spectrum cross-correlated with itself (auto-correlation), each of the EPA solid supplements (EPAS) (EPAS1-EPAS5), and EPA liquid supplements (EPAL). Only EPAS5 appears to be significantly different. One-class modeling of the cross-correlated spectra of the average EPA with each of the individual EPA samples (plot not shown) showed that the EPAS and EPAL spectra, with the exception of EPAS5 were the same as the cross-correlated EPA population. These results provide some interesting conclusions. First, since the correlation spectra for EPAS1-EPAS4, and EPAL were similar, the supplements contained similar extracted components. This means that the starting ingredients and the processes used by the manufacturers were similar. Second, since the auto-correlation spectrum of EPA was similar to the correlation spectra of EPA with EPAS1-EPAS4 and EPAL, the reference samples in this study were similar to the raw ingredients used by the manufacturers. Finally, since the extracted compounds were the same, the analytical extraction process used in this study was similar to the preparation method employed by the manufacturers. These data also indicate that supplement EPAS5 was produced using either a different starting ingredient or a different extraction process.

**FIGURE 13:**
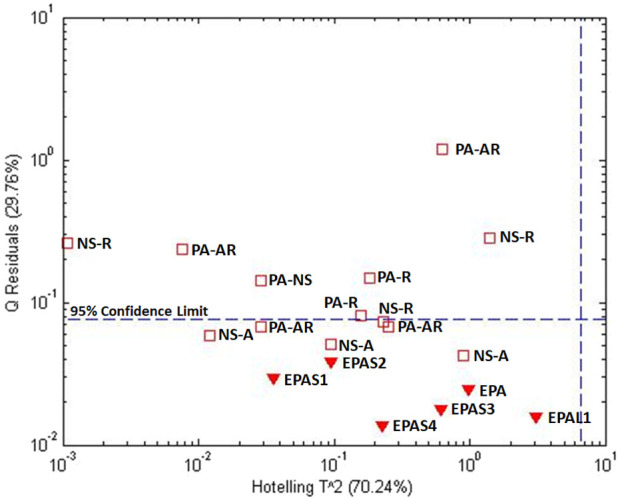
Influence plot of mixed supplements spectra cross correlated with the average *E. purpurea* single ingredient spectra based on EPA, EPAS, and EPAL (Figure 12). Labels: EPA – *E. purpurea* aerial, EPAS1-EPAS4 – EPA solid supplements, EPAL - EPA liquid supplement. Mixed labels have two factors. First factor abbreviations are NS - species no specified, P – *E. purpurea*, A- *E. angustifolia*. Second factor abbreviations are NS – plant part not specified, A – aerial, and R – root.

Figure 13 shows a one-class model for mixed supplements based on the cross-correlated spectra for EPA, EPAS1-EPAS4, and EPAL. All mixed supplements that were known to contain aerial plant material fell within the 95% confidence level, even if the species were not specified. All supplements with root material, even if the species were not specified, fell outside the 95% confidence limit. The exceptions falling outside the 95% confidence limit were supplements composed of both species and both plant parts. This suggests that perhaps the aerial portion was of insufficient concentration to provide a clear cross-correlated spectrum.

The data in Figures 12, 13 indicate that cross correlation of supplements with the raw ingredient accurately identifies ions found in both spectra. Ions common to both spectra provide an enhanced multiplicative product whereas ions found in only one spectrum provide a product close to zero. Thus, cross-correlation and one-class modeling can identify commonalities of the raw ingredients and supplements.

#### Maca (*Lepidium meyenii* walpers)

Maca data were presented earlier for the general description of one-class modeling. This study was a collaboration with the American Botanical Council; Gaia Herbs; Hong Kong Baptist University; Charles Sturt University & Therapeutic Research, TTD International Pty Ltd; and NSF International Authenticity Laboratory (Geng et al., 2020). There is some question as to whether the plant material is accurately identified as *L. meyenii* Walpers or *Lepidium Peruvianum* Chacon (Meissner et al., 2015). Samples consisted of 39 commercial maca supplements from 11 manufacturers, 31 unprocessed maca roots grown in Peru and China, and an historic non-tuber maca sample from Peru. Samples were analyzed using FIMS and DNA next-generation sequencing (NGS).

Initial, untargeted PCA placed all the maca samples in 3 classes: commercial maca samples, roots grown in Peru, and roots grown in China (Figure 3A). With one-class modeling the commercial Maca was readily differentiated from the raw root materials (Figures 3C, D). A similar approach, selecting either the Chinese or Peruvian samples for the model, showed that either could be differentiated from the other two classes (data not shown).

**FIGURE 14:**
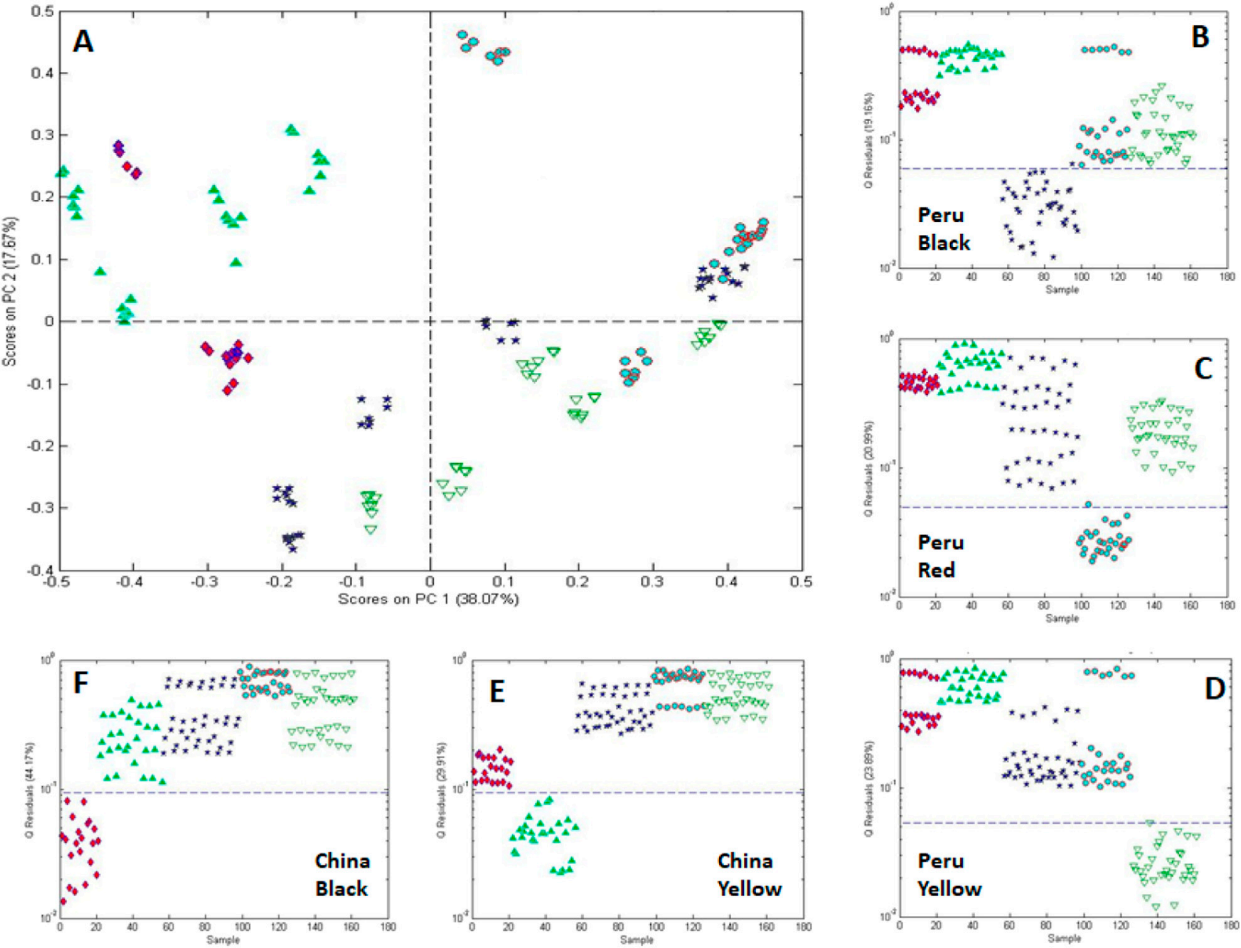
One-class modeling of FIMS fingerprints acquired by negative ionization after processing by multivariate ANOVA showing **(A)** the score plot for black, red, and yellow maca acquired from Peru and China surrounded by **(B–F)** single-class models for each of the 5 country-color combinations. Sample icons: 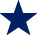 Peru black, 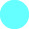 Peru red, 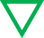 Peru yellow, 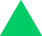 China yellow, and 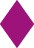 China black.

One-class modeling was also combined with analysis of variance (ANOVA) to differentiate roots based on country (China and Peru) and color (black, red, and yellow) (Figure 14). ANOVA was used to isolate the mean variance for each experimental factor (country, processing, and color) and cross factor to provide residuals free of the variance of the other factors (Harnly et al., 2014). One-class modeling of the individual factor residuals provided plots in Figures 14B–F. These data show, not surprisingly, that the metabolite composition correlates with country and color.

Metabolite profiling using ultra-high performance liquid chromatography-high resolution mass spectrometry (UHPLC-HRMS) in combination with PCA loadings was used to annotate the compounds responsible for differentiating between country and color. Genetically, all samples were confirmed to be similar and to be L. meyenii Walpers based on NGS at 3 gene regions (ITS2, psbA, and trnL) and comparison to recorded sequences of vouchered standards.

The results of this study show that one-class modeling can be used in combination with ANOVA to determine the statistical significance of differences arising from experimental factors associated with the samples. The metadata associated with genetics, country of origin, year of origin, climate, and handling can have a major impact on a plant’s metabolite profile and one-class modeling provides a versatile in deconvoluting the interaction of the factors.

## Conclusion

One-class modeling is a versatile method that can be readily applied to any set of multivariate data (targeted or non-targeted) data. This is a supervised method (reference samples must be identified) that develops a model based on the characteristics of a single class of samples, the reference samples. Using PCA, the Q statistic offers an inherent combined metric which can be used to determine if the reference and test samples belong to the same class, i.e., the test sample is authentic or adulterated. It can also be used as an effective tool for determining the similarity of sample metabolites for different species and, with cross-correlation, the similarity of raw ingredients and supplements. Finally, in combination with ANOVA, one-class modeling can be used to determine the significance of experimental factors. The beauty of one-class modeling is that it can be implemented on any commercial chemometrics platform and is applicable to any data file, i.e., chromatograms, spectra, or database, targeted or non-targeted.
